# Ropivacaine and lidocaine-induced central nervous system toxicity following brachial plexus block in two uremic patients

**DOI:** 10.1097/MD.0000000000043242

**Published:** 2025-07-04

**Authors:** Manhua Zhu, Yong Qi, Song Zhang, Jingwen Zhou

**Affiliations:** a Department of Anesthesiology, The Affiliated LiHuiLi Hospital of Ningbo University, Zhejiang, China.

**Keywords:** brachial plexus block, central nervous system, lidocaine, ropivacaine, toxicity

## Abstract

**Rationale:**

Ropivacaine and lidocaine are amide local anesthetics (LAs) commonly used for brachial plexus block, and with lower central nervous system (CNS) toxicity and cardiovascular toxicity than bupivacaine. However, emerging evidence suggests that neurotoxicity associated with these LAs may still occur, particularly in high-risk populations such as uremic patients, who may exhibit altered drug metabolism and increased susceptibility to adverse effects. Therefore, we aimed to report and analyze 2 cases of CNS toxicity following ropivacaine and lidocaine administration in this population. The findings will contribute to a better understanding of the safety profile of these LAs in uremic patients.

**Patient concerns:**

We describe 2 cases of CNS toxicity in uremic patients following brachial plexus blocks induced by a total of 20 mL 0.375% ropivacaine mixed with 1% lidocaine. Both patients became unconscious and failed to respond to verbal after brachial plexus blocks. However, their vital signs were stable and no signs of cardiovascular toxicity were observed.

**Diagnoses:**

The 2 patients were diagnosed with CNS toxicity following brachial plexus block. The diagnosed of CNS toxicity was based on:

(1) Unconscious and failed to respond to verbal after LAs injection.

(2) Exclusion of alternatives: no hypoglycemia; normal arterial blood gases; absence of focal neurological signs suggesting stroke or epilepsy.

(3) Supporting factors: reduced renal clearance may lead to larger plasma concentrations of LAs in uremic patients; hypertension likely resulted from sympathetic activation during CNS toxicity.

**Interventions:**

Both patients were given oxygen via a mask at a rate of 3 L/min, standard monitoring, and close observation. Arterial blood gas analysis was immediately conducted.

**Outcomes:**

They were both fully conscious 2 hours after the brachial plexus block. Vascularized injection sites, uremic status, preexisting cardiac dysfunction, and the mixing of the 2 local anesthetics may be the reasons for the occurrence of the CNS toxicity.

**Lessons:**

When performing peripheral nerve block, it is necessary to identify whether there are risk factors that increase the incidence of local anesthetic systemic toxicity.

## 1. Introduction

Ballon angioplasty for arteriovenous fistula stenosis is generally performed using regional anesthesia or general anesthesia. Patients with chronic kidney disease (CKD) often have more comorbidities, such as hypertension, anemia, and hyperkalemia. The limitations with general anesthesia are increased cardiovascular and respiratory risks during the perioperative period and delayed postoperative recovery.^[1]^ Moreover, it was reported that the failure rates of vascular accesses may be reduced under regional anesthesia compared to general anesthesia.^[2]^ Hence, peripheral nerve block is becoming more and more popular among patients undergoing such surgery.

Ropivacaine and lidocaine are amide local anesthetics (LAs) with lower central nervous system (CNS) toxicity and cardiovascular toxicity than bupivacaine. However, reports of neurotoxic accidents caused by ropivacaine and lidicaine are not uncommon. CNS toxicity associated with ropivacaine ranges widely from 20 to 400 mg.^[3]^ In clinical practice, LA are administered within defined maximum recommended doses. The maximum recommended doses of lidocaine and ropivacaine for brachial plexus block are 300 and 225 mg, respectively.^[4]^ In this report, we describe 2 cases of CNS toxicity following ultrasound-guided brachial plexus blocks (interscalene and axillary approaches) using standard doses of ropivacaine and lidocaine in uremic patients.

## 2. Case report

Written informed consent was obtained from both patients and legal representatives.

### 2.1. Case 1

**Figure 1.**
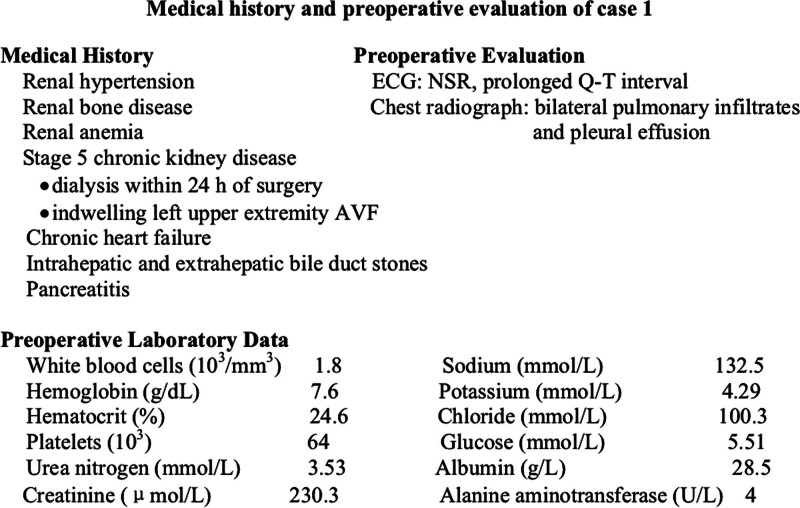
Medical history and preoperative evaluation of case 1. AVF = arteriovenous fistula, ECG = electrocardiogram, NSR = normal sinus rhythm.

The patient was a 68-year-old, 41-kg, 150-cm female, who was scheduled for ballon angioplasty for left upper extremity arteriovenous fistula stenosis. Her medical history and preoperative evaluation were shown in Figure 1. After entering the operating room, the patient was monitored with a 5-lead electrocardiography, pulse oximetry (SpO_2_), and noninvasive arterial blood pressure. A peripheral IV line was established and 0.9% saline was administered. Her blood pressure (BP) was 161/70 mm Hg, heart rate (HR) was 59 bpm, and SpO_2_ showed 95% saturation. Oxygen was administered at 3 L/min via nasal cannula. Ultrasound-guided interscalene and axillary brachial plexus blocks were performed with an in-plane technique. A linear probe (6–13 MHz, Edge, Sonosite, Seattle) was used to identify the anterior and middle scalene muscle, and the C5–7 nerve roots of brachial plexus. After disinfection, a 5-cm, 22-gauge needle was used to perform a interscalene brachial plexus block with a posterior-to-anterior trajectory. A total of 10 mL LA (0.375% ropivacaine mixed with 1% lidocaine) was administered after repeated negative aspiration. Subsequently, the axillary brachial plexus block was performed. The linear probe was placed perpendicular to the long axis of the arm, as the axillary artery, median, ulnar, radial, and musculocutaneous nerves were identified, a total of 10 mL the same LA as before was administered around these nerves after repeated negative aspiration.

After the procedure, she didn’t experience any discomfort and her vital signs were stable. Fifteen minutes later, the patient became unconscious and failed to respond to verbal and painful stimulus. Her BP was 182/113 mm Hg, HR was 78 bpm, and SpO_2_ decreased to 87%. Three liters per minute of mask oxygen was administered immediately. Arterial blood gas analysis was then conducted and revealed a low oxygen partial pressure (pH: 7.4, PaO_2_: 62.2 mm Hg, PaCO_2_: 39.6 mm Hg, HCO_3_^‐^: 22.6 mmol/L, K^+^: 4.0 mmol/L, Na^+^: 131.0 mmol/L, Cl^‐^: 103.0 mmol/L, Glu: 8.5 mmol/L). Then, SpO_2_ gradually rose to 98%.

An hour and a half later, she gradually regained consciousness. Her BP fluctuated between 180/95 and 200/120 mm Hg, and her HR was between 70 and 98 bpm. She was fully awake and oriented 2 hours after brachial plexus block. The surgery was canceled and she was transferred to the intensive care unit for close observation. The next day, she was returned to the nephrology ward. A week later, she received the scheduled surgery under general anesthesia without any adverse reactions.

### 2.2. Case 2

**Figure 2.**
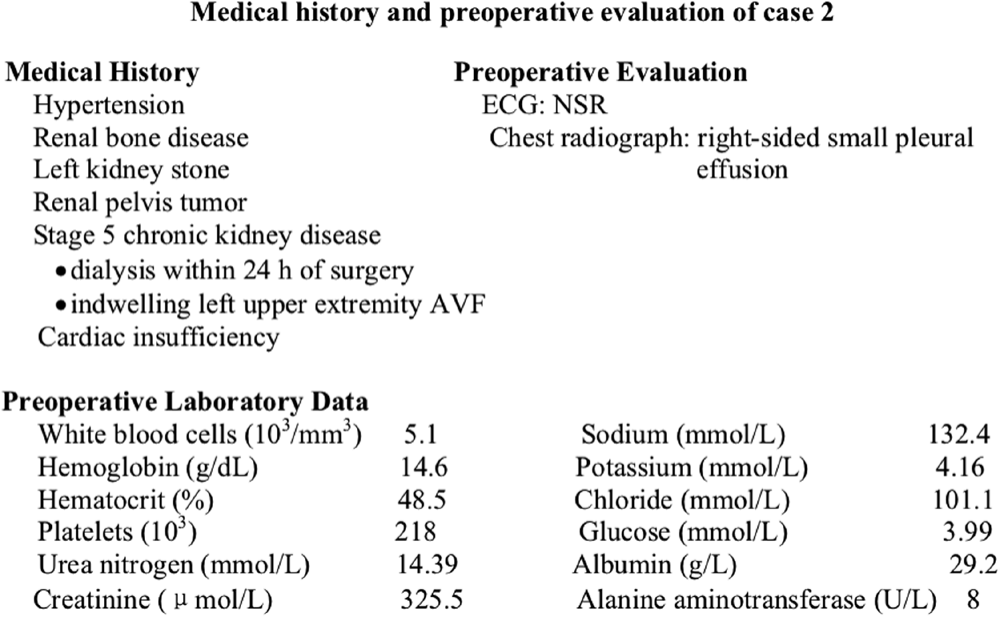
Medical history and preoperative evaluation of case 2. AVF = arteriovenous fistula, ECG = electrocardiogram, NSR = normal sinus rhythm.

The patient was an 81-year-old, 52-kg, 160-cm female, who was scheduled for the same surgery as in case 1. Her medical history and preoperative evaluation were shown in Figure 2. After entering the operating room, she received standard monitoring, IV infusion of 0.9% saline, and 3 L/min of nasal cannula oxygen, as in case 1. Her BP was 190/93 mm Hg, HR was 85 bpm, and SpO_2_ showed 99% saturation. She only received an ultrasound-guided interscalene brachial plexus block. And the nerve block procedure was the same as that in case 1. After repeated negative aspiration, a total of 10 mL the same ropivacaine/lidocaine solution as in case 1 was injected in anterior to the brachial plexus, then withdrew the needle to the posterior surface of the brachial plexus and an additional 10 mL of ropivacaine/lidocaine was administered.

After the procedure, she did not experience any discomfort and her vital signs were stable. Ten minutes later, the interscalene block was tested, a complete sensory and motor block of the left arm had developed. Then, the surgery began and lasted for a total of 45 minutes. During the surgery, her BP fluctuated between 166/85 and 185/92 mm Hg, HR was between 80 and 92 bpm, and SpO_2_ was between 97% and 100%. Throughout the surgery, the patient kept quiet and closed her eyes to rest.

After the surgery, the anesthesiologist found that the patient was unable to be fully aroused. However, the patient’s vital signs were stable, and arterial blood gas analysis showed no abnormalities (pH: 7.4, PaO_2_: 137.9 mm Hg, PaCO_2_: 39.3 mm Hg, HCO_3_^‐^: 22.8 mmol/L, K^+^: 3.5 mmol/L, Na^+^: 133.5 mmol/L, Cl^‐^: 102.6 mmol/L, Glu: 3.9 mmol/L). Then, she was given oxygen via a mask at a rate of 3 L/min, standard monitoring, and close observation. She was fully awake 2 hours after brachial plexus block. Fifteen minutes after regaining consciousness, she was sent back to the nephrology ward.

## 3. Discussion

Both patients presented with unconsciousness and unresponsiveness to verbal stimuli following brachial plexus block, consistent with CNS toxicity of local anesthetic systemic toxicity (LAST). However, the following differential diagnoses were considered and ruled out:

### 3.1. Hypoglycemia

Rapid-onset altered mental status can occur in uremic patients due to impaired gluconeogenesis or insulin clearance.

Ruling out: Blood glucose levels were checked immediately and were within normal range (case 1: 8.5 mmol/L; case 2: 3.9 mmol/L).

### 3.2. Cerebrovascular event (stroke/seizure)

Uremic patients are at higher risk of stroke due to accelerated atherosclerosis and platelet dysfunction.

Ruling out: No focal neurological deficits (e.g., hemiparesis, facial droop) were observed. No postictal confusion or tonic–clonic movements suggestive of seizure.

### 3.3. Hypoxia or hypercapnia

Respiratory depression due to high interscalene block affecting the phrenic nerve could lead to hypoventilation.

Ruling out: Except for the transient decrease of SpO_2_ in case 1, SpO_2_ remained > 95% after oxygen inhalation, and arterial blood gas showed normal PaCO_2_ levels.

### 3.4. Metabolic encephalopathy (uremia/hyperammonemia)

CKD patients may develop uremic encephalopathy, but this usually progresses more gradually.

Ruling out: No prior signs of confusion or asterixis. Arterial blood gas showed no severe metabolic acidosis.

Given the temporal relationship with LA injection, absence of alternative explanations, and rapid recovery (within 2 hours, consistent with LA metabolism), CNS toxicity due to LAST was considered.

The clinical presentation of LAST varies from dizziness, agitation, seizures to neurological depression, respiratory and heart failure, and 32% of patients had an isolated CNS effects.^[5]^ CNS toxicity in the form of loss of consciousness is the less common clinical manifestation of LAST. It was reported that approximately half of the neurologic or cardiac toxicity after LA injections occurred within 10 minutes, 19% happened between 11 minutes and 1 hour.^[5]^ We report 2 cases of loss of consciousness occurring more than 10 minutes after injection of ropivacaine and lidocaine in 2 uremic patients. Both patients exhibited CNS inhibition after brachial plexus block, but did not show any symptoms of cardiovascular depression. And the intraoperative BP of both patients was relatively high. The possible reasons are as follows: (1) The uremic patients had hypertension before the operation. (2) Sympathetic stimulation caused by rising LA concentrations after brachial plexus block resulted in BP elevation.

Ropivacaine, when used for brachial plexus block in a dose range of 2.25 to 4.5 mg/kg, shows no evidence of CNS toxicity.^[6]^ Lidocaine is the safest LA, the maximum recommended dose for lidocaine without adrenaline is 4.5 mg/kg.^[7]^ In these 2 cases, we used 75 mg ropivacaine mixed with 200 mg lidocaine, which were far below the maximum recommended doses. Considering that lidocaine offers the fastest onset but the lowest duration of action, while ropivacaine has a slower onset but a longer duration, we chose to use a mixture of the 2 LAs.

Considering the similar clinical feature of CNS toxicity in these 2 cases, we speculate 4 possible explanations. First, despite repeated negative aspirations and direct injection under ultrasound guidance did not rule out the possibility of an inadvertent intravascular injection, the late onset of the CNS symptom suggests rapid absorption of ropivacaine and lidocaine into circulation in our 2 cases. Patients with uremia often exhibit a state of hyperdynamic circulation, which can quickly flush the deposits of LAs.^[8]^ The neck and armpit are areas richly vascularized combined with hyperdynamic circulation, making these 2 patients at high-risk for LAST. Second, the plasma concentration of LAs was higher in uremia patients. It was reported that patients with uremia have larger total plasma concentration of ropivacaine and its main metabolites after brachial plexus block compared to those without uremia.^[9]^ Ropivacaine and lidocaine are highly bound to α_1_-acid glycoprotein (AAG), which is increased in uremic patients and provides protection against LAST. However, reduced renal clearance of LA and the metabolites may lead to larger plasma concentrations of LA in uremic patients.^[9]^ Unfortunately, we did not measure the plasma levels of ropivacaine and lidocaine in these 2 patients. Due to the lack of plasma LA level monitoring, our diagnosis relies predominantly on clinical judgment. Third, both patients were elderly and suffered from cardiac insufficiency. Patients with preexisting heart disease, extremes of age and frailty are at-risk groups predisposed to LAST.^[5]^ Fourth, both patients were administered ropivacaine and lidocaine, which are both amide LAs, and their toxicities are additive. Considered together, vascular-rich injection sites, uremic status, preexisting cardiac dysfunction, and the mixture of 2 LAs may each contribute to increasing patients’ susceptibility to the CNS toxicity of lower dosage LAs.

In patients with severe renal impairment, the terminal elimination half-life of ropivacaine is approximately 2.59 hours.^[10]^ Both patients recovered 2 hours after LAST may be related to this factor. The elevated plasma AAG levels associated with uremia may have prevented more severe complications in these 2 cases. In addition, since neither of these 2 patients exhibited severe cardiovascular toxicity, and considering that CKD can lead to changes in lipid metabolism and cause dyslipidemia,^[11]^ lipid emulsion was not administered.

There was no cardiac toxicity in both cases. Previous studies indicate that CNS symptoms occur at lower plasma LA concentrations than cardiac toxicity.^[12]^ Our patients likely received subthreshold cardiotoxic doses due to: (1) The dosages of LA used in both patients did not exceed the maximum recommended dose. (2) Fractional administration limits peak plasma levels. (3) The strong AAG binding of ropivacaine and lidocaine in uremic patients reducing free toxic fractions. Besides, compared with bupivacaine, ropivacaine has lower cardiotoxicity.^[13]^

This study has several limitations: First, the diagnosis of LAST was primarily based on clinical presentation rather than direct measurement of plasma ropivacaine and lidocaine concentrations. Second, the small sample size (only 2 cases) limits the generalizability of our findings. Third, the potential influence of uremia-related pharmacokinetic changes on LAST susceptibility remains incompletely understood, and future studies should incorporate pharmacokinetic assessments.

## 4. Conclusion

This case report highlights 2 rare cases of isolated CNS toxicity following brachial plexus block with ropivacaine and lidocaine in uremic patients, despite LAs doses administered was lower than recommended doses. The delayed onset of symptoms suggests rapid systemic absorption, and may be exacerbated by uremia-related hyperdynamic circulation, altered pharmacokinetics, and vascular-rich injection sites. These cases highlight the increased susceptibility of uremic patients to LAST, even with conventional doses, and emphasize the need for: (1) Vigilant monitoring for delayed CNS toxicity in this population. (2) Cautious LA dosing in elderly or frail patients with renal impairment. (3) Further pharmacokinetic studies to clarify the optimal recommended dose of LA for uremia.

## Author contributions

**Conceptualization:** Manhua Zhu.

**Data curation:** Manhua Zhu, Song Zhang, Jingwen Zhou.

**Writing – original draft:** Manhua Zhu.

**Writing – review & editing:** Manhua Zhu, Yong Qi.

## References

[1] Siracuse JJ Gill HL Parrack I. Variability in anesthetic considerations for arteriovenous fistula creation. J Vasc Access. 2014;15:364–9. 24811604 10.5301/jva.5000215

[2] Jorgensen MS Farres H James BLW. The role of regional versus general anesthesia on arteriovenous fistula and graft outcomes: a single-institution experience and literature review. Ann Vasc Surg. 2020;62:287–94. 31382001 10.1016/j.avsg.2019.05.016

[3] Rodolà F Anastasi F Vergari A. Ropivacaine induced acute neurotoxicity after epidural injection. Eur Rev Med Pharmacol Sci. 2007;11:133–5. 17552143

[4] Rosenberg PH Veering BT Urmey WF. Maximum recommended doses of local anesthetics: a multifactorial concept. Reg Anesth Pain Med. 2004;29:564–75; discussion 524. 15635516 10.1016/j.rapm.2004.08.003

[5] Macfarlane AJR Gitman M Bornstein KJ El-Boghdadly K Weinberg G. Updates in our understanding of local anaesthetic systemic toxicity: a narrative review. Anaesthesia. 2021;76(Suppl. 1):27–39. 10.1111/anae.15282 33426662

[6] Ala-Kokko TI Löppönen A Alahuhta S. Two instances of central nervous system toxicity in the same patient following repeated ropivacaine-induced brachial plexus block. Acta Anaesthesiol Scand. 2000;44:623–6. 10786752 10.1034/j.1399-6576.2000.00522.x

[7] Klein JA Jeske DR. Estimated maximal safe dosages of tumescent lidocaine. Anesth Analg. 2016;122:1350–9. 26895001 10.1213/ANE.0000000000001119 PMC4830750

[8] Neff MS Kim KE Persoff M Onesti G Swartz C. Hemodynamics of uremic anemia. Circulation. 1971;43:876–83. 4931294 10.1161/01.cir.43.6.876

[9] Pere P Salonen M Jokinen M Rosenberg PH Neuvonen PJ Haasio J. Pharmacokinetics of ropivacaine in uremic and nonuremic patients after axillary brachial plexus block. Anesth Analg. 2003;96:563–9, table of contents. 12538213 10.1097/00000539-200302000-00048

[10] Pere PJ Ekstrand A Salonen M. Pharmacokinetics of ropivacaine in patients with chronic renal failure. Br J Anaesth. 2011;106:512–21. 21307007 10.1093/bja/aer002

[11] Moradi H Vaziri ND. Molecular mechanisms of disorders of lipid metabolism in chronic kidney disease. Front Biosci (Landmark Ed). 2018;23:146–61. 28930541 10.2741/4585

[12] Weinberg GL Palmer JW VadeBoncouer TR Zuechner MB Edelman G Hoppel CL. Bupivacaine inhibits acylcarnitine exchange in cardiac mitochondria. Anesthesiology. 2000;92:523–8. 10691241 10.1097/00000542-200002000-00036

[13] Santos AC Arthur GR Pedersen H Morishima HO Finster M Covino BG. Systemic toxicity of ropivacaine during ovine pregnancy. Anesthesiology. 1991;75:137–41. 2064038 10.1097/00000542-199107000-00022

